# Supplementary figures and images for: Relief from nitrogen starvation entails quick unexpected down-regulation of glycolytic/lipid metabolism genes in enological Saccharomyces cerevisiae
Source: PLoS One. 2019 Apr 25;14(4):e0215870. doi: 10.1371/journal.pone.0215870 (PMC6483528; doi:10.1371/journal.pone.0215870)

a

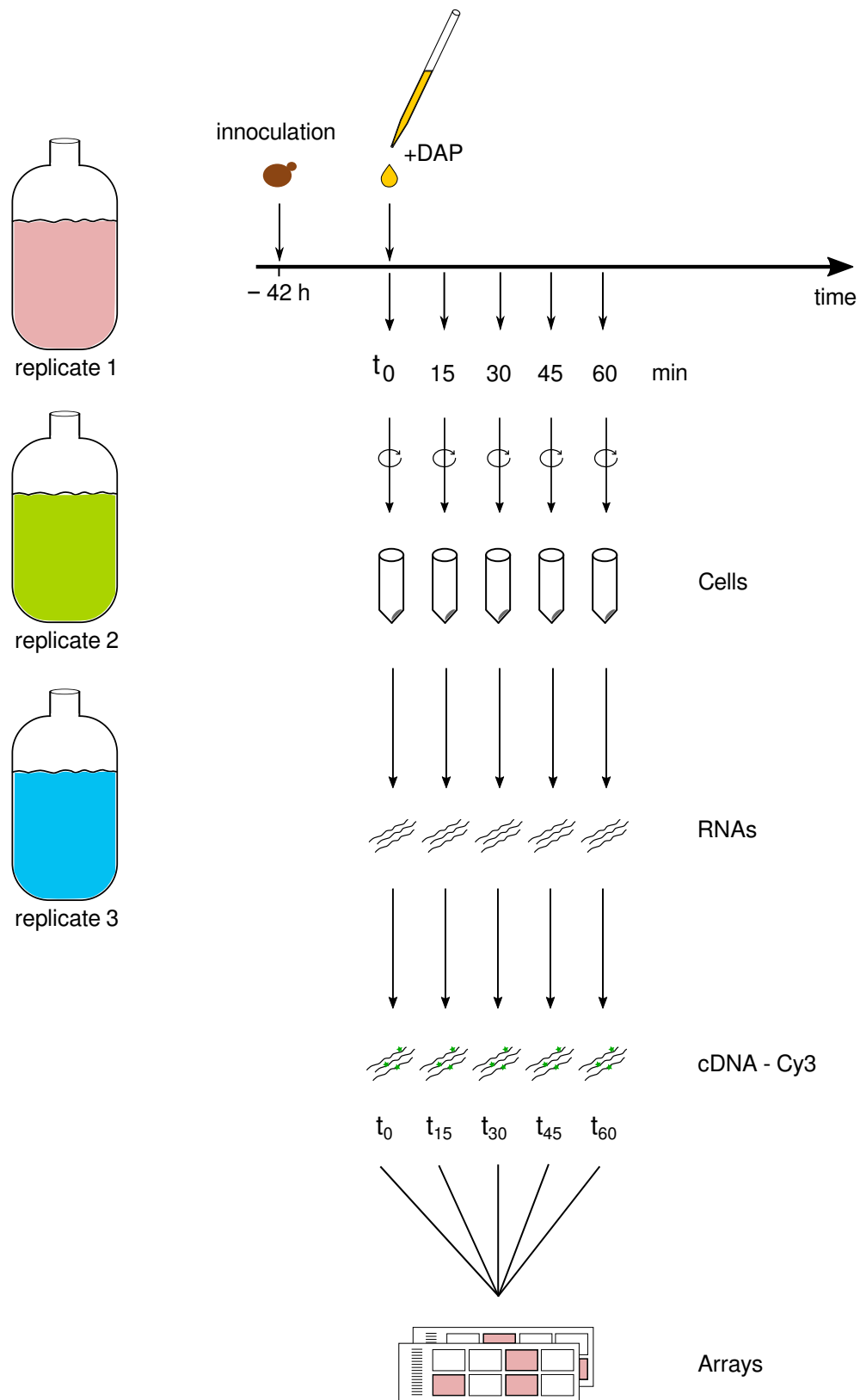

b

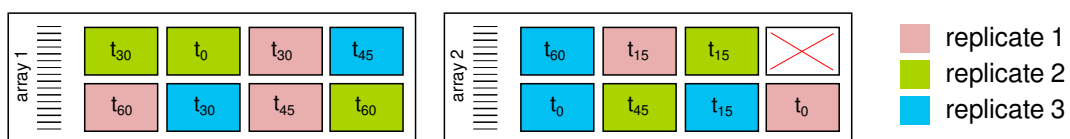

Supplement: S1 Fig — Schematic representation of the experimental design. (PDF) [file pone.0215870.s002.pdf]
