# Supplementary material for: Relief from nitrogen starvation entails quick unexpected down-regulation of glycolytic/lipid metabolism genes in enological Saccharomyces cerevisiae
Source: PLoS One. 2019 Apr 25;14(4):e0215870. doi: 10.1371/journal.pone.0215870 (PMC6483528; doi:10.1371/journal.pone.0215870)
Supplement: S2 Fig — Schematic representation of methods used to analyze the expression data: selection of a model for the time-course experiment, step-regression and manual clustering of expression profiles. (PDF) [file pone.0215870.s003.pdf]

S2 Figure

$$Y = b_0 + b_1 t + b_2 t^2 + \varepsilon$$

model of gene expression over time

Step 1

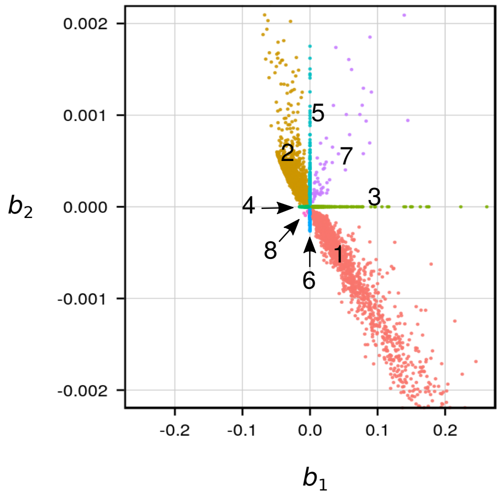

estimation of model coefficients by step regression

cluster identification

Step 2

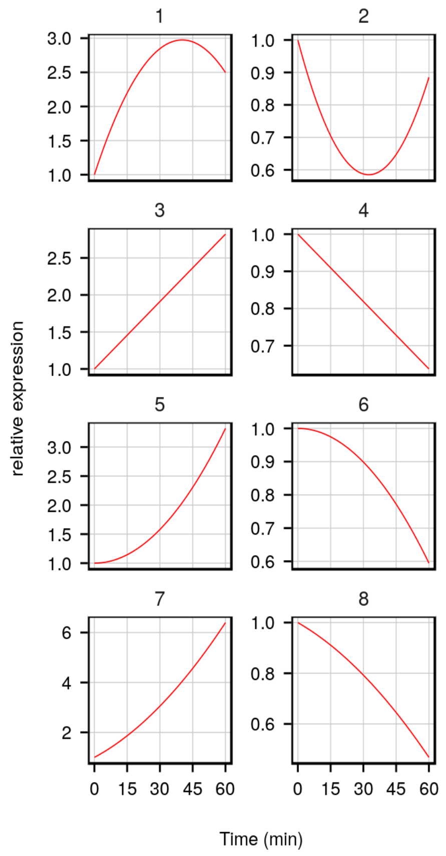

average expression per cluster

Step 3
